# The distribution of the inferior hypogastric plexus in female pelvis

**DOI:** 10.25122/jml-2022-0145

**Published:** 2022-06

**Authors:** Ofelia-Costina Goidescu, Iulian-Alexandru Dogaru, Theodor-Georgian Badea, Mihaly Enyedi, Octavian Enciu, Daniela-Elena Gheoca Mutu, Florin-Mihail Filipoiu

**Affiliations:** 1 Doctoral School, Carol Davila University of Medicine and Pharmacy, Bucharest, Romania; 2 Discipline of Anatomy, Faculty of Medicine, Carol Davila University of Medicine and Pharmacy, Bucharest, Romania; 3 Department of Surgery, Faculty of Medicine, Carol Davila University of Medicine and Pharmacy, Bucharest, Romania

**Keywords:** inferior hypogastric plexus, hypogastric nerves, pelvic autonomous nervous system, mesorectum

## Abstract

Elements that comprise the inferior hypogastric plexus are difficult to expose, intricate, and highly variable and can easily be damaged during local surgical procedures. We aimed to highlight, through dissection, the origin, formation, and distribution of the hypogastric nervous structures and follow them in the female pelvis. We performed detailed dissections on 7 female formalin-fixed cadavers, focusing on structures surrounding the pelvic organs. For each hemipelvis, we removed the peritoneum from the pelvic floor, and after we identified the hypogastric nerves, we continued our dissection towards the inferior hypogastric plexuses, following the branches of the latter. Laterorectally, the hypogastric nerves form the inferior hypogastric plexus, a variable structure – nervous lamina, neuronal network (more frequently), or sometimes a combination of them. We identified three components of the inferior hypogastric plexus. The anterior bundle travels towards the base of the urinary bladder, the middle part innervates the uterus and the vagina, and the posterior segment provides the innervation of the rectum. The plexus can be identified after removing the pelvic peritoneum and the subperitoneal adipose tissue. Intraoperatively, the structures can be preserved by using an immediately-subperitoneal dissection plane. The variable branches are relatively well-organized around the pelvic vessels, supplying the urinary bladder, the genital organs, and the rectum. The ureter is surrounded by some branches, especially in its last segment, and it also receives innervation directly from the hypogastric nerve. Close to the viscera, the nerves enter neurovascular plexuses, making the intraoperative separation of the nerves and the vessels virtually impossible.

## INTRODUCTION

The prevertebral sympathetic plexuses generate a preaortically-located nervous network with well-known ganglionic stations – the caeliac, superior mesenteric, renal, and inferior mesenteric ganglia. The preaortic nervous network is continued inside the pelvic cavity by the superior hypogastric plexus. This plexus then divides into the two hypogastric nerves, which end up, bilaterally, into a complex nervous network known as the inferior hypogastric plexus [1]. This plexus presents a variable and complex structure and gives off its branches as groups of fascicles heading towards the pelvic organs. The inferior hypogastric plexuses receive a parasympathetic component through the sacral nerves and a sympathetic one through anastomoses with the presacral sympathetic chain ganglia. The modern surgical approach for abdominopelvic pathologies is based upon specific dissections, with the conservation of the nervous dependencies of the hypogastric plexuses [2]. Thus, the sexual, glandular and sphincter functions are left intact. If, however, the inferior hypogastric plexuses are damaged during surgery, the patients may experience loss of erection (penile or clitoral) and the capacity to orgasm, as well as urinary and fecal incontinence. For example, in the case of the radical hysterectomy, a meta-analysis by Lee et al. in 2019 evaluated data from 23 articles that assessed the frequency of inferior hypogastric plexus injury in two types of surgeries – nerve-sparing radical hysterectomy (NSRH) and conventional radical hysterectomy (CRH). The authors reported that, in the case of NSRH, there were 6/493 colorectal dysfunctions (events/number of surgeries), 9/93 sexual dysfunctions, 57/767 urinary dysfunctions. For CRH, 40/532 colorectal dysfunctions, 8/129 sexual dysfunctions, and 132/738 urinary dysfunctions were reported [3].

In this context, we aimed to identify the superior hypogastric plexus, the hypogastric nerves, and the inferior hypogastric plexuses and to observe how the branches of the latter reach the pelvic organs. The subperitoneal localization of the plexuses makes the identification of the nervous structures somewhat counterintuitive. We believe that the demonstration, through anatomical dissection, of how the hypogastric plexuses are organized and the identification of useful intraoperatively landmarks can be excellent instruments in adapting and updating the surgical protocols worldwide in order to preserve the respective nervous structures.

## MATERIAL AND METHODS

We performed dissections on seven female cadavers aged between 65 and 74 years old, with no previous surgical history, that were preserved using 9% concentration formalin solution, in the dissection hall of the Discipline of Anatomy, at Carol Davila University of Medicine and Pharmacy, Bucharest, Romania. The anatomization of the corpses was done according to national legislation and university regulations. We sectioned the pelvises in the midsagittal plane. Through dissection, we demonstrated the hypogastric plexuses and their branches. For each of the dissected hemipelvises, we removed the pelvic peritoneum and, following the identification of the hypogastric nerves, continued the dissection of the inferior hypogastric plexus by applying traction on the hypogastric nerves to facilitate the dissection.

Consequently, we followed the branches of the inferior hypogastric plexus toward the pelvic organs. The dissection results were photographed as we concentrated our efforts on the areas of interest around the main organs in the pelvis. We kept some of the branches of the internal iliac artery and the ureter to observe the disposition of the nervous structures in relation to them. All the resulting images were edited without altering the scientific content.

## RESULTS

**Figure 1:**
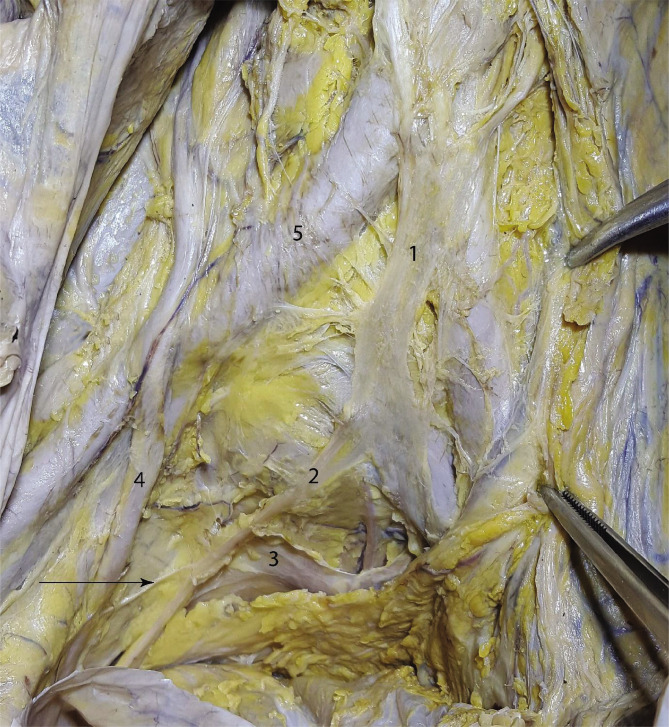
The superior hypogastric plexus – visible after the removal of the peritoneum. 1 – Superior hypogastric plexus; 2 – Right hypogastric nerve; 3 – Presacral fascia; 4 – Right ureter; 5 – Right common iliac artery. Black arrow points toward a ureteric branch from the right hypogastric nerve. The forceps hold the peritoneum.

We cut out a triangular peritoneal flap along the course of the common iliac arteries that we subsequently folded towards the anterior. The superior hypogastric plexus is immediately subperitoneal and presacral and shaped as a nervous lamina, from which small branches emerge towards the common iliac arteries. At the level of the aortic bifurcation, two preaortic nervous trunks (left and right), coming from the inferior mesenteric plexus and the preaortic plexus, unite and form the superior hypogastric plexus. The superior hypogastric plexus is shaped as a lamina of approximately 4×1.5 cm, as seen in Figure 1, and it descends retroperitoneally, on the midline, superficial to the presacral fascia and the middle sacral artery. The plexus can either be compact or have the aspect of a network. The superior hypogastric plexus has lateral relations with the common iliac arteries and, further, with the two ureters. The peritoneum is somewhat difficult to detach from the superior hypogastric plexus, and the latter is visible after removing the subperitoneal connective-adipose tissue. Inferiorly, the superior hypogastric plexus is continued by the two hypogastric nerves, right and left. These nerves remain attached to the peritoneum and can be exposed by pulling the peritoneum in order to keep it in tension. During rectal resection surgeries, for example, the surgeon can gain a cleavage plane that is found between the mesorectal fascia and the peritoneum, posterolateral to the rectum. This plane is where the hypogastric nerves descend into the pelvis, going superficial to the branches of the internal iliac arteries, and the nerves must be spared at this point during the surgery. This is possible if the operator applies traction to the peritoneum. We followed the trajectory of the hypogastric nerves towards the inferior until we reached the inferior hypogastric plexus.

**Figure 2:**
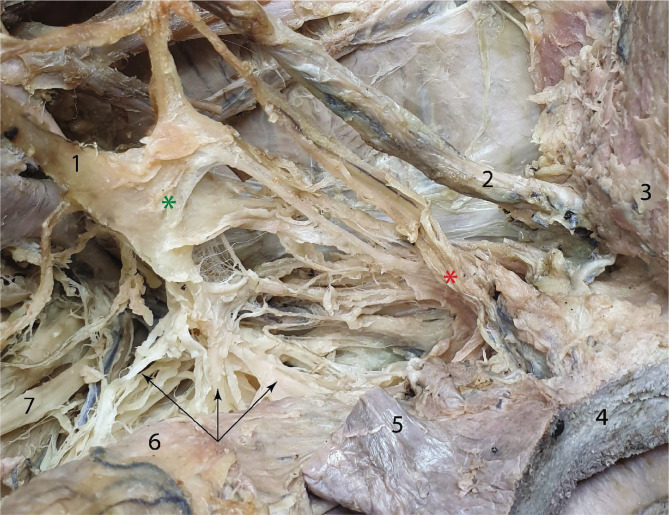
Overview of the left inferior hypogastric plexus, located subperitoneal, on the pelvic floor – after the resection of arterial branches. 1 – Hypogastric nerve; 2 – Ureter; 3 – Urinary bladder; 4 – Uterus; 5 – Fallopian tube; 6 – Rectum covered by the rectal fascia; 7 – S4 sacral nerve. Green asterisk – inferior hypogastric plexus; red asterisk – branches supplying the uterus; black arrows – rectal branches spreading around the organ.

The dissection of the inferior hypogastric plexus and the identification of its branches is difficult because the inferior hypogastric plexus is filled with adipose-connective tissue that adheres to the neurovascular structures. This makes their intraoperative identification strenuous. Laterorectally, close to the internal iliac artery, the hypogastric nerves form the inferior hypogastric plexuses. The inferior hypogastric plexus can have a variable structure, as it can appear either as a nervous lamina, a nervous network, or a combination of the two. We identified the three divisions of the plexus´ branches which are anterior, intermediate, and posterior. The anterior bundle innervates the base of the urinary bladder, the intermediate one supplies the uterus and the vagina, and the posterior component enters the mesorectum and innervates the rectum. In Figure 2, we can observe a nervous lamina located superiorly, from which three bundles of nervous fibers emerge inferiorly, directed toward the pelvic organs. A well-represented nervous trunk emerges from the infero-posterior angle of the nervous lamina, supplying the rectum. The rectal branches emerge in a fan-like manner and advance towards the rectum, which they approach from the anterior, lateral, and posterior, respectively. From the infero-anterior angle, the lamina gives off another bundle of nerves that go towards the uterus and the urinary bladder. These branches will join the uterine artery and the ureter on their path toward the respective organs.

**Figure 3:**
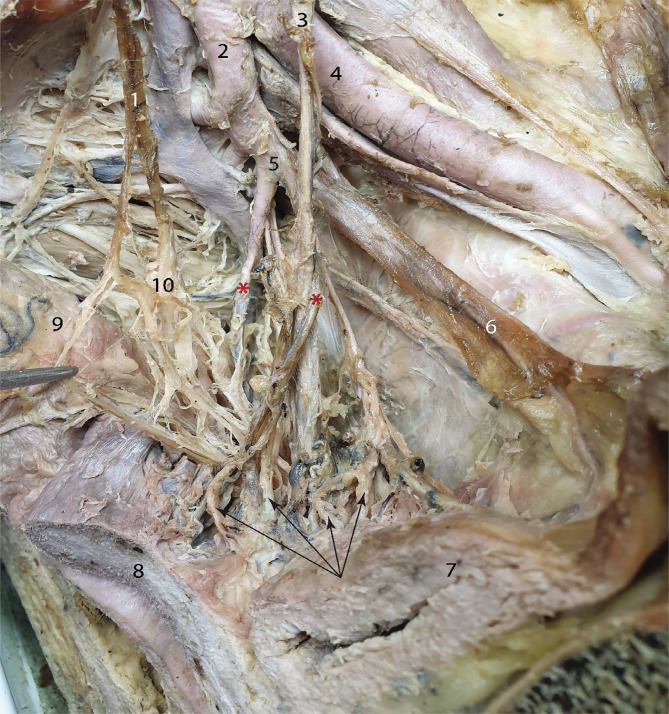
The distribution of the nervous branches in the utero-vesical angle, left side. 1 – Hypogastric nerve; 2 – Internal iliac artery; 3 – Ureter; 4 – External iliac artery; 5 – Common trunk for the two uterine arteries; 6 – Superior vesical artery; 7 – Urinary bladder; 8 – Uterus; 9 – Rectum; 10 – Inferior hypogastric plexus. Red asterisks – two uterine arteries; black arrows – branches of the inferior hypogastric plexus that run into the utero-vesical angle.

We would like to point out that the inferior hypogastric plexus branches are closely related to the vesical and uterine vessels. In Figure 3, two uterine arteries can be observed. Only the anterior artery presents the "classical" relation with the ureter. This artery is also the source of the inferior vesical artery and ends up at the level of the utero-vesical space, deep to the utero-vesical peritoneal fold. The posterior uterine artery is directed towards the base of the broad ligament of the uterus. The voluminous nervous bundle that emerges from the anterior angle of the inferior hypogastric plexus enters the utero-vesical space and arrives at the two organs. The inferior branches that stem from this bundle reach the erectile organs. In the utero-vesical space, the nervous bundle is joined by arteries and veins. The uterine branches are grouped around the anterior uterine artery, whereas the vesical branches travel along the ureter and the inferior vesical artery. The nerves approach the urinary bladder at the level of the base and of the lateral border.

**Figure 4:**
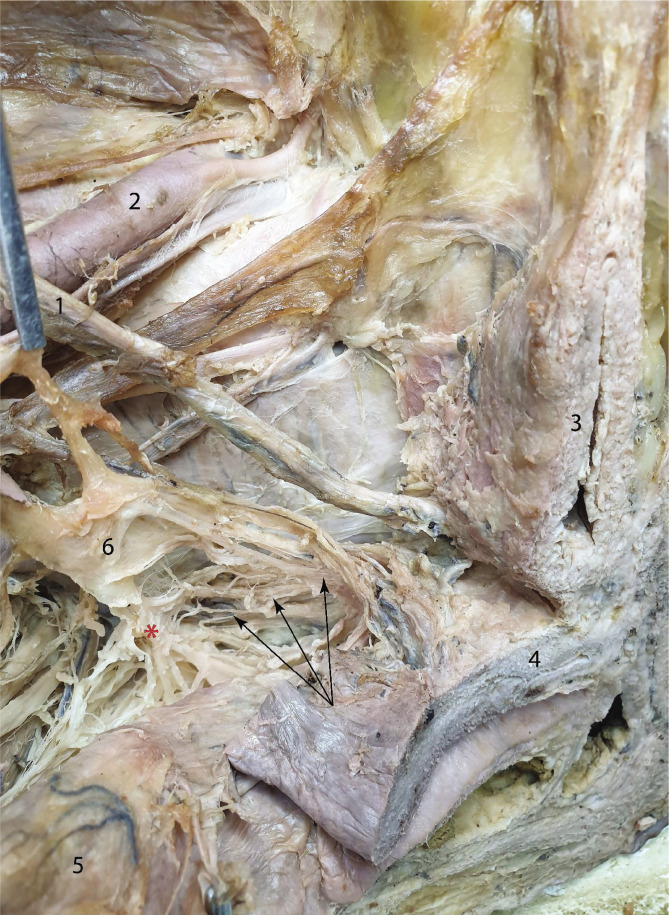
Showcasing the uterine branches of the inferior hypogastric plexus. 1 – Ureter; 2 – External iliac artery; 3 – Urinary bladder; 4 – Uterus; 5 – Rectum; 6 – Inferior hypogastric plexus. Red asterisk – rectal division; black arrows – uterine branches.

We separated the branches that supply the uterus from the anterior bundle of fibers that emerge from the anterior angle of the inferior hypogastric plexus. They approach the uterus from antero-lateral, as visible in Figure 4.

**Figure 5:**
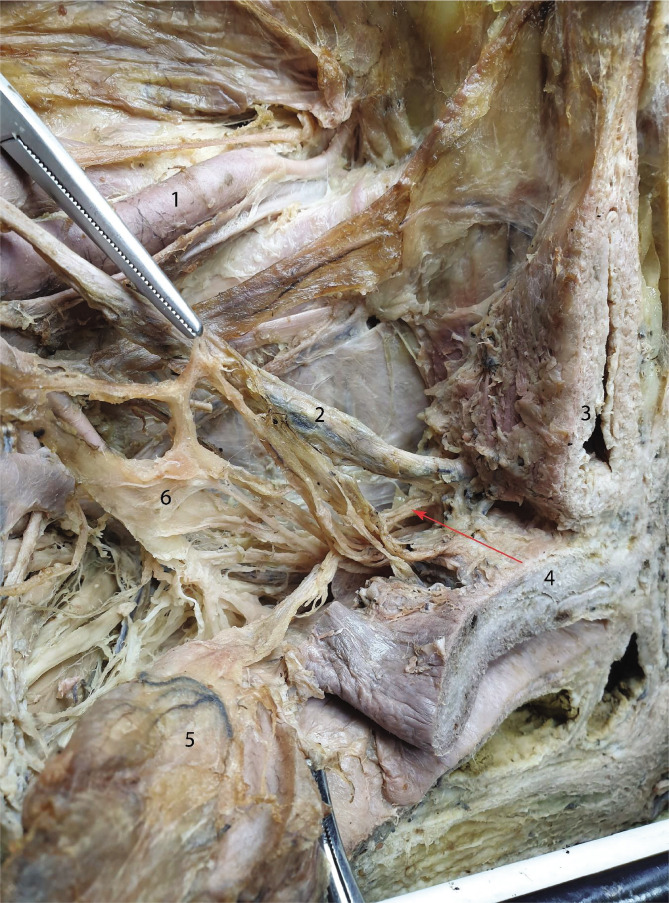
Showcasing the vesical branches of the inferior hypogastric plexus. 1 – External iliac artery; 2 – Ureter; 3 – Urinary bladder; 4 – Uterus; 5 – Rectum; 6 – Inferior hypogastric plexus. Red arrow points towards vesical branches that join the last segment of the ureter. The upper forceps holds vesical branches.

The nerves that supply the urinary bladder arrive from three directions. As previously shown in Figure 1 as well, the hypogastric nerve gives off thin branches that join the ureter on its way toward the bladder. Another group of nerves originating in the antero-superior angle of the inferior hypogastric plexus takes a periureteric pathway. Lastly, a smaller group of deep branches join the ureter along its last 2 centimeters before entering the urinary bladder, as shown in Figure 5.

**Figure 6:**
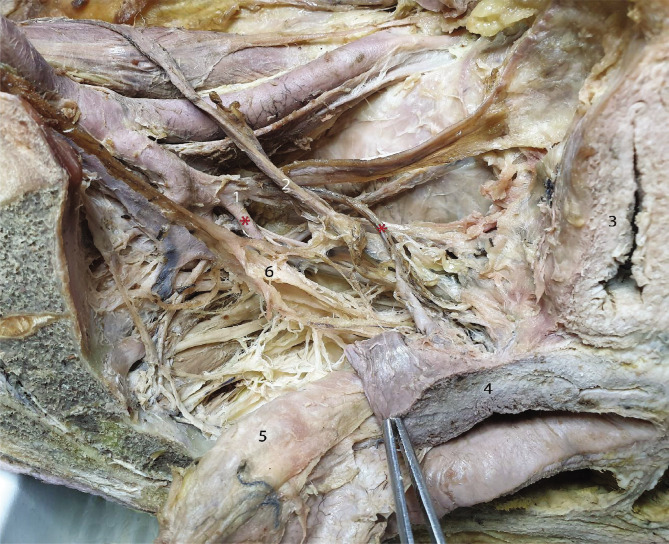
Overview of the distribution of the inferior hypogastric plexus branches along the uterine arteries and the ureter. 1 – Common trunk for the two uterine arteries, highlighted with red asterisks; 2 – Ureter; 3 – Urinary bladder; 4 – Uterus; 5 – Rectum; 6 – Inferior hypogastric plexus.

Figure 6 is an overview of the left hemipelvis, which facilitates the local orientation, as the sacral plexus branches, sacral sympathetic chain, (double) uterine artery, and the bifurcation of the common iliac artery are visible. Thus, we demonstrated anatomical landmarks that are useful in the identification of the inferior hypogastric plexus and its branches.

## DISCUSSION

The dissection of the inferior hypogastric plexus is laborious since the nervous elements are hard to expose and closely related to the pelvic organs and vessels. The identification of the plexus is only possible after the removal of the pelvic posterior parietal peritoneum and of the subperitoneal adipose-connective tissue, medial to the plane of the internal iliac vessels [4].

The plexus has either the aspect of a nerve fiber network or that of a compact lamina of nervous tissue [5]. The branches are relatively well-organized around the blood vessels that supply the pelvic viscera. Intraoperatively, lesions involving the nervous structures can be avoided if the surgeon uses an immediate subperitoneal dissection plane [6, 7]. The surgeon must apply traction to the peritoneum to expose the correct dissection plane [8].

After they emerge from the superior hypogastric plexus, the hypogastric nerves adopt a divergent trajectory on the pelvic floor, still in relation to the peritoneum [9]. The nerves remain attached to the peritoneum during the anatomical and surgical dissection [10, 11].

The branches of the inferior hypogastric plexus are numerous and highly variable, but they can be organized in three main streams – anterior, middle, and posterior, for the urinary bladder, genitalia, and rectum [12, 13]. The ureter is surrounded by the anterior branches of the plexus, especially in the last part of its pathway [14–16]. The same fiber disposition also applies to the uterine artery [17]. We demonstrated that it does receive, though, direct branches from the hypogastric nerve. The anatomical variation is substantial in terms of position, thickness, aspect, and manner of ramification. Close to the target organs, the nerves enter neurovascular plexuses, and the intraoperative separation of the nervous and the vascular structures is virtually impossible.

The modern approach to pelvic surgery must take into consideration the patients´ quality of life, which relies on the integrity of the nervous structures responsible for urinary continence and sexual function. Thus, pelvic surgery is bound to strive to achieve better results, not only in terms of oncological safety but also in what concerns the functional outcomes. This is possible by using pelvic dissection as a training instrument and a source of information.

## CONCLUSIONS

Because of its role, position, and complex anatomy, the plexus can be damaged during regional surgical procedures such as Wertheim's radical abdominal hysterectomy, rectal resection surgery, prostatectomy, and cystectomy and, essentially, any abdominal surgery that involves the pelvic viscera. Thus, the knowledge of the anatomy of the inferior hypogastric plexus is crucial for the general surgeon, the urologist and the gynecologist. Moreover, our research demonstrates the importance of the anatomical study in itself, which allows the surgical specialist to observe better the different variants of the intricate structures of the hypogastric plexus.
